# Manfred Eigen: the realization of his vision of Biophysical Chemistry

**DOI:** 10.1007/s00249-017-1266-y

**Published:** 2017-12-11

**Authors:** Herbert Jäckle, Carmen Rotte, Peter Gruss

**Affiliations:** 1 Max Planck Institute for Biophysical Chemistry, Am Faßberg 11, 37077 Göttingen, Germany; 2 Present Address: Okinawa Institute of Science and Technology, 1919-1 Tancha, Onna, Kunigami, Okinawa 904-0495 Japan

**Keywords:** Manfred Eigen

## Abstract

Manfred Eigen turned 90 on May 9th, 2017. He celebrated with a small group of colleagues and friends on behalf of the many inspired by him over his lifetime—whether scientists, artists, or philosophers. A small group of friends, because many—who by their breakthroughs have changed the face of science in different research areas—have already died. But it was a special day, devoted to the many genius facets of Manfred Eigen’s oeuvre, and a day to highlight the way in which he continues to exude a great, vital and unbroken passion for science as well as an insatiable curiosity beyond his own scientific interests. He continues to dismiss arguments such as, that scientific problems cannot be solved because of a current lack of appropriate tools, or because of the persuasion of the community that certain things are immeasurable. He has lived up to and accepted only the highest scientific standards with his fundamental contributions in widely differing research fields, for which he has received numerous prizes and honorary doctorates, including the Nobel Prize for Chemistry in 1967. Some of his outstanding contributions to science and technology are honored in the following chapters. Here, we will report some characteristic traits of Manfred Eigen, and his personal development. We highlight his visionary foresight regarding how multidisciplinary science should combine to study the complex processes of life and its evolution in establishing an institute that applied biological, chemical, and physical methods, and how his vision became sustained reality.

## Science or music?

Manfred Eigen grew up in a musical family. Concerts and piano music were a characteristic part of his childhood. At pre-school age, he started to play the piano and practiced intensively, but after a few years, he felt that his enthusiasm for playing music faded, and admitted so to his father, a professional cellist of the Bochum Symphony Orchestra. Much to his surprise, his father accepted his wish, but imposed two conditions. Firstly, he should give up piano playing entirely because tinkling would not be tolerable for his father. Secondly, he should devote the time he would have spent practicing piano playing to other meaningful activities. To the concern of his mother, he established a small laboratory at home, which he extensively used for experimentation: “It was a real laboratory, which my mother was not at all happy with, especially when something exploded yet again”, Manfred Eigen remembered, and so it was initially no problem for him to obey his father’s precept.

However, Manfred Eigen’s passion for music was greater than he thought when making the agreement with his father, possibly also because he was constantly surrounded by music in his parents’ home where chamber concerts regularly took place. He met well-known artists not only at official concerts, but closely and personally at home. These events increasingly strengthened his desire to continue with music, and he secretly started again practicing to finally surprise his father with a special birthday present—to play Schubert’s Arpeggione Sonata together with him. His father was so impressed by his son’s talent that he insisted on sending him to a first-rate teacher for piano lessons. Manfred Eigen did what he always does—he brought his piano playing to perfection. As a 12-year-old child, he gave public performances including piano concerts by Bach, Haydn, and Dittersdorf. His future was predetermined towards a career as a pianist!

The Second World War interrupted his dreams. Fifteen-year-old Manfred Eigen had to serve in an anti-aircraft unit with no chance to practice piano playing. When he returned from the war, he therefore decided to follow his second passion—to the great benefit of science as it turned out later—and matriculated at the University in Göttingen, where he became a full-time student of physics. There was only little time left to practice piano for some years until he again devoted time to active piano playing in the late 50s by taking lessons from Rudolf Hindemith, the younger brother of Paul Hindemith, and his wife Maria Landes-Hindemith. Many who participated in workshops and conferences with Manfred Eigen experienced his delightful piano interludes. Two of his piano concerts of Mozart, accompanied by the *New Orchestra of Boston* under David Epstein and the *Basler Kammerorchester* under the direction of Paul Sacher, are immortalized on CD.

In one of his concerts in Basel, Sydney Brenner was in the audience, sitting next to a professional pianist. Sydney asked what she thought about the quality of Manfred Eigen’s performance. “Not bad for a chemist” was the answer. That evening, Manfred Eigen told Sydney his ideas about the hypercycle, which he developed together with Peter Schuster, and his quasispecies model. “Not bad for a pianist…” was Sydney’s reaction. Not bad…!

Together with his friend Paul Sacher, Manfred Eigen tried to convince the Max Planck Society’s leadership to establish a kind of “*Musik*-*Bauhaus*”, which would connect research in art and science by bringing together renowned scientists and musicians in one institute. This idea was preceded by the “*Hinterzartener Kreis*”, where he and leading natural scientists of different research areas—including Werner Heisenberg, Carl Friedrich von Weizsäcker, and Konrad Lorenz, the philosophers Georg Picht and Theodor Adorno as well as musicians like the violinist Yehudi Menuhin, the composer and conductor Pierre Boulez, and the flutist Aurèle Nicolet—met to discuss the concept. This project failed, since the Max Planck Society decided that Manfred Eigen’s idea should not be realized. Therefore, no surprise, he smiled when the society discussed much later his initial idea again and subsequently founded the Max Planck Institute (MPI) for Empirical Aesthetics in Frankfurt in 2012. Manfred Eigen’s proposal failed, fortunately for us, because he then devoted all his efforts to establish our institute, the MPI for Biophysical Chemistry.

This brings us back to his scientific roots and the university years of Manfred Eigen. As already mentioned, due to the lack of practice, Manfred Eigen gave up the idea of becoming a professional musician. When he returned from the war, after managing to escape from an American prisoner-of-war camp near Salzburg (Austria), he walked all the way to Göttingen to enroll there in physics and chemistry in 1945. The University of Göttingen was one of the first German universities to open up again after the Second World War. The young student immediately had contact with outstanding scientists: he attended physics lectures by Werner Heisenberg and Wolfgang Paul; the former was already a Nobel Laureate, the latter would become one.

He completed his diploma thesis with Arnold Eucken, who was so impressed by his outstanding abilities that he proposed that Manfred Eigen should enroll immediately as a doctoral student. He effortlessly measured up to the expectations placed upon him. He was only 24 years of age when he successfully completed his PhD in physical chemistry, subsequently becoming a research assistant at the Institute for Physical Chemistry at the University of Göttingen.

### Measuring immeasurably fast reactions

“The rate of true neutralization reactions has proven to be immeasurably fast”. Manfred Eigen had found this quote in Arnold Eucken’s chemistry textbook *Lehrbuch der chemischen Physik* while preparing for his PhD examination. This book was his “bible of physical chemistry”, but he was then of an age at which one accepts practically nothing without asking critical questions. So he started to reflect on just how fast an immeasurably fast reaction might be. In 1953, he accepted a position as an assistant at the MPI for Physical Chemistry, with Karl Friedrich Bonhoeffer as a supervisor and mentor. He turned his attention to the study of extremely fast chemical reactions, focusing on “the immeasurable”.

At that time, chemical reaction rates could be measured down to a thousandth of a second. Convinced that nothing in chemistry was immeasurable and that the problem was simply a matter of unsuitable experimental tools, Manfred Eigen successfully began to develop the so-called relaxation measurement methods. He was fortunate: Leo De Maeyer, who later became a director at the new MPI for Biophysical Chemistry, joined his group and it turned out that he was essential for developing the necessary equipment for measuring ultrafast reactions. The approach involves the perturbation of a system in chemical equilibrium, by sound wave, for example, to then measure the time the system requires to return to its original state of equilibrium. Due to this “trick”, the “immeasurable” became measurable. Manfred Eigen presented his results to the British *Faraday Society* in 1954, showing that this method made it possible to determine reaction rates at the micro- and nanosecond scale—a scientific sensation! Manfred Eigen’s method solved key issues not only in physical chemistry but also in biochemistry, for example, by allowing an understanding of how enzyme activities are controlled.

In 1958, Manfred Eigen was appointed a Scientific Member of the Max Planck Society. Four years later he became head of the Department of *Chemical Kinetics* at the MPI for Physical Chemistry, and was appointed as director in 1964. His laboratory in Göttingen attracted chemists from all over the world who wanted to investigate ultrafast reactions.

Just about 10 years after measuring the immeasurable, Manfred Eigen’s major scientific breakthrough was honored with the Nobel Prize in Chemistry, which he received together with Ronald G. W. Norrish and George Porter in 1967.

## Setting up an institute devoted to multidisciplinary life sciences

Nowadays, it is undisputed that research in life sciences should be multidisciplinary, involving physics, chemistry, biology, and mathematics. Already in 1968, Manfred Eigen offered the Max Planck Society ideas for establishing a new interdisciplinary institute. Initially, the Max Planck Society was somehow skeptical. However, Manfred Eigen was successful, possibly because of his internationally recognized achievements. Also, most likely his Nobel Prize helped to convince the leadership and, in fact, he was not short of offers to continue his research at renowned research institutions all over the world. No matter for what reason, the Max Planck Society offered Manfred Eigen the opportunity to build this new institute by fusing two MPIs in Göttingen, the MPI for Physical Chemistry and the MPI for Spectroscopy, to find a place for its location, and to design it both in architectural (together with his colleague Manfred Kahlweit) and scientific terms, before leading the new institute as a permanent “managing director”. He turned down the last part of the offer and headed only the *Department of Biochemical Kinetics* until he retired in 1995. His personal modesty, his respect for his colleagues, and his way of solving problems with the best factual and transparent solution instead of imposing his unquestioned authority shaped the spirit of the institute, which he devoted to his mentor: Karl Friedrich Bonhoeffer. Officially, the institute opened its doors in 1971.

One year after having been awarded the Nobel Prize in Chemistry, Manfred Eigen turned his attention with his usual intensity to the problem of self-organization of matter and the evolution of biological macromolecules. While investigating reaction mechanisms of biochemical processes, he had been fascinated time and again by the optimal efficiency and precision of the molecular interactions in biology. However, he was not satisfied with a purely phenomenological explanation such as optimum adaptation as put forward by Darwin. He put Darwin’s idea of evolution by means of natural selection on a firm physical footing and applied it to molecular systems, thereby establishing a fundamental bridge between biology and physics. The concepts of *hypercycle*, *quasispecies*, *error threshold*, and *sequence space* are inseparably linked to his name. Furthermore, fascinated by the idea of establishing a multidisciplinary institute that studies these complex processes as well as the molecular evolution of life using biological, chemical, and physical methods, three new directors were recruited in addition to the six directors who joined Manfred Eigen’s new institute at its foundation. The first nine departments were designated to *Biochemical Kinetics* (Manfred Eigen), *Experimental Methods* (Leo De Maeyer), *Molecular Biology* (Thomas Jovin), *Kinetics of Phase Transitions* (Manfred Kahlweit), *Electrochemistry and Reaction Kinetics* (Hans Strehlow) and *Spectroscopy* (Albert Weller) of the MPIs for Physical Chemistry and for Spectroscopy. Three new departments were added by external recruitment, namely *Neurobiology* (Otto D. Creutzfeldt), *Molecular Systems* (Hans Kuhn), and *Laser Physics* (Fritz Peter Schäfer).

Obviously, in the beginning the institute was still heavy on physical chemistry-related research, but soon after and over the years, a second generation of departments addressing directly biological problems were added to the institute, including *Neurochemistry* (Victor Wittaker), *Biochemistry and Cell Biology* (Klaus Weber), *Membrane Biophysics* (Erwin Neher), *Cell Physiology* (Bert Sakmann), *Molecular Genetics* (Dieter Gallwitz), *Molecular Cell Biology* (Peter Gruss), *Spectroscopy and Photochemical Kinetics* (Jürgen Troe) as well as *Molecular Developmental Biology* (Herbert Jäckle). Manfred Eigen guided the establishment of these new departments according to his philosophy: “It is not the research area that counts, it is the excellence of the individuals”.

Currently, the third generation of department directors has been appointed according to the same philosophy, still focusing in his spirit on the biological questions and the necessary tool development: once a department director retires, and continues as emeritus as Manfred Eigen did himself, new recruits are not selected on the basis of “best scientific fit” to ongoing research but by asking the question of whether the person would add excellence to the institute. And—also in the spirit of Manfred Eigen—young investigators are given the chance to develop and follow their own ideas during a transient stay of up to 9 years at the institute without the possibility of getting a tenured position. However, exceptions are the rule and actually, several of the very best young investigators have been offered the chance to stay, being promoted by the Max Planck Society to department directors at the institute. This practice is based on the insight that the best people should not leave the institute, and it has turned out to be a successful strategy: scientists of each of the by now three generations of directors at the institute have been awarded the Nobel Prize—Manfred Eigen himself, Erwin Neher, and Bert Sakmann of the second generation and Stefan Hell of the third generation. Each of them started a career as a research group leader at the institute.

Currently, the institute hosts 13 departments including *Neurobiology* (Reinhard Jahn), *Cellular Biochemistry* (Reinhard Lührmann), *NMR*-*based Structural Biology* (Christian Griesinger), *Genes and Behavior* (Gregor Eichele), *Cellular Logistics* (Dirk Görlich), *NanoBiophotonics* (Stefan Hell), *Molecular Developmental Biology* (Herbert Jäckle), *Theoretical and Computational Biophysics* (Helmut Grubmüller), *Physical Biochemistry* (Marina Rodnina), *Dynamics at Surfaces* (Alec Wodtke), *Molecular Biology* (Patrick Cramer), *Meiosis* (Melina Schuh) as well as *Structural Dynamics* (Holger Stark). In addition, the institute continues to support young investigators by hosting 23 research groups as a springboard for the careers of junior researchers. In setting up the institute, Manfred Eigen—despite his strong love for theoretical biology—applied his leitmotif: “In biology, pure theory—in the absence of experimental results—proves to be poor theory”, a motto of the institute that has been maintained for almost half a century.

## Another proposal that failed: unfortunately!

The many creative ideas of Manfred Eigen included the proposal that the Max Planck Society should establish an “Institute for Theoretical Biology” by combining theory and modeling with corresponding experiments, as in part encompassed by the research done at the institute he successfully established. This proposal was put forward in the early 1990s, before the Institutes for Systems Biology were established in Seattle (USA) and Tokyo (Japan). It was also before systems biology emerged as a movement in its own right, and also in advance of the claim of the US National Science Foundation, which put forward a grand challenge for systems biology in the 21st century to build a mathematical model of the whole cell. Manfred Eigen’s postulation was based on his foresight of holistic approaches such as genome projects, the large increase in data from the *omics* and the accompanying advances in high-throughput experiments as well as bioinformatics and an era of “big data”, which is coming now. It is possible that the Max Planck Society again missed a chance by not participating in the foresight of an intellectual giant!

## Conflating ideas

“You must be hungry for science”—this attitude has enabled Manfred Eigen to make a formative impact on the lives of many staff and research colleagues in “his institute” and beyond, who are unanimous in their enthusiasm about the inspiration and unselfish support which he has always been prepared to give. “You must communicate!” Communication also meant to him explaining the secrets of science to the public, may it be through TV shows or by his famous book “Das Spiel” (English version: “Laws of the Game: How The Principles of Nature Govern Chance”, 1983) which he wrote together with his life-long scientific partner and second wife, Ruthild Oswatitsch-Eigen. It also includes his latest book “From Strange Simplicity to Complex Familiarity. A Treatise on Matter, Information, Life and Thought.” which he published—again with the help of his wife Ruthild—in 2013.

More than anything else, Manfred Eigen liked to listen to the latest results during the weekly “Teestunde” (tea hour) in his department, and he immediately got to the strong and weak points no matter which subject was reported. His own lectures reflected not only his wide range of interests and broad spectrum of knowledge, but also his enormous enthusiasm for research, which he communicated to his audiences. The *Molecular Biology Colloquium* founded by him together with Fritz Cramer in Göttingen, and the *Winter Seminar* Eigen established in 1966, became traditions. What started out as a departmental meeting involving a small circle of people turned into an ever-increasing meeting in the years that followed: Renowned scientists from all over the world, including more than 50 Nobel Laureates to date, are among those who have taken part in the legendary “Winter Seminar” in Klosters, Switzerland—surroundings much loved by Manfred Eigen, a passionate skier and hiker. And—of course—as a passionate musician he quite often turned the meeting room in Klosters or the close by abbey church into a “concert hall” where the multi-talented scientist performed a musical interlude.

## Founder of evolutionary biotechnology

“Everything which is new has to come out of fundamental research, otherwise it’s not new”. Manfred Eigen has not only discovered new things but has also applied them to create new products. In fact, his theories on the self-organization of complex molecules and his development of evolution machines, in which he put these theories into practice, gave rise to a new branch of biotechnology—“evolutionary biotechnology”. The evolution machines, developed to a level of industrial applicability by Manfred Eigen’s team at the institute, were used successfully to investigate basic mechanisms of evolution at high speed in the laboratory. They were used to learn and exploit the tricks of, for example, the AIDS virus and other pathogens to outwit the immune system. Such evolution machines were moreover employed in single molecule detection to help in finding new active substances and to use them for drug development. Evolutionary biotechnology is being taken over and applied by companies co-founded by Manfred Eigen, such as Evotec AG and DIREVO Biosystems, which became later part of Bayer HealthCare AG.

## Leaving active science without losing curiosity and impact

Manfred Eigen retired in 1995 but continued with his research both in Göttingen and at the Scripps Research Institute in La Jolla (California, USA). The Scripps Research Institute became a second home for Manfred Eigen to which he was attracted to by his friend Richard Lerner, the president of the institute at the time. After finishing his latest book in 2013, Manfred Eigen retired from participating in meetings because he was not in the best of health. However, he continues to curiously and hungrily follow the latest publications and he loves to listen to the latest results obtained at “his” institute in Göttingen. And, as always, he critically points out what is missing in order to convince him of the conclusions: non-aggressive but distinct, generally sprinkled with his humor. Beyond science, he continues to give advice to his colleagues at the institute, insinuating what could or should be planned and handled in a better way. Not as a teacher, but as a friend, a quality that has molded the togetherness of the faculty and shaped the spirit of the institute. His integrating and modest personality set a sustainable standard of how colleagues interact at the institute. This is to highlight that Manfred Eigen not only served as a scientific role model, but he also set the standard for the social intercourse among colleagues and for the next generation(s) of scientists at the institute.

Because of his numerous achievements and highly acknowledged publications in different fields of science, Manfred Eigen acquired the reputation of being one of the most versatile German researchers. Bochum, the city of his birth, named him as Honorary Citizen of the University, and the city of Göttingen awarded him an honorary citizenship. His scientific prizes, awards, and honors are numerous, acknowledging not only his impact on various scientific fields but also underlining his decisive impetus that was in a big way responsible for advancing science beyond the founding and fostering of the MPI for Biophysical Chemistry. To name a few activities, he served as Chairperson of the EMBO Council and as Chairperson of the Scientific Advisory Board of the Basel Institute for Immunology (Switzerland). In the 11 years he served as president of the *Studienstiftung des deutschen Volkes* (*German Academic Scholarship Foundation*), he demonstrated his great commitment to the next generation of scientists and established doctoral grants for the very best. Having Manfred Eigen as a mentor, both for scientists and for the institute, was a blessing. His vision, successfully realized under his guidance, as well as his integrative way of interacting with people, imprinted his spirit at the MPI for Biophysical Chemistry, a spirit which remains a permanent challenge for the institute to live up to.

